# Antiretroviral therapy (ART) management of Low-Level Viremia in Taiwan (ALLEVIATE)

**DOI:** 10.7448/IAS.17.4.19785

**Published:** 2014-11-02

**Authors:** Chien-Yu Cheng, Yu-Zhen Luo, Pei-Ying Wu, Wen-Chun Liu, Shan-Ping Yang, Jun-Yu Zhang, Shu-Hsing Cheng, Chien-Ching Hung

**Affiliations:** 1 Department of Infectious Diseases, Taoyuan General Hospital, Tao-Yuan, Taiwan; 2 Department of Infectious Diseases, National Taiwan University Hospital, Taipei, Taiwan; 3 Department of Infectious Diseases, National Taiwan University College of Medicine, Taipei, Taiwan

## Abstract

**Introduction:**

This retrospective study aimed to investigate that if switch of combination antiretroviral therapy (cART) would result in viral suppression (<40 copies/mL) at 48 weeks for patients with persistent low-level viremia after having received cART for six months or more at two hospitals designated for HIV care in Taiwan.

**Materials and Methods:**

Between January 2001 and January 2013, patients were enrolled if plasma HIV RNA load (PVL) were >20 to <1000 copies/mL detected for six months or more [1, 2]. Using a standardized data collection form, we recorded data of PVL and CD4 count before cART and at the detection of low-level viremia, serologies for hepatitis B and C virus, risk factors, duration of cART exposure, years of HIV diagnosed and ever experiencing treatment failure. The strategy of switch is based on the clinical guidelines of BHIVA, which suggest change of cART from non-nucleoside reverse-transcriptase inhibitors (nNRTIs) or unboosted protease inhibitor (PI) to boosted PI, newer boosted PI or ARV of different mechanism [3].

**Results:**

In this study, 165 patients were enrolled, 119 patients (72.1%) did not switch (Group 1), and 46 patients (27.9%) switched previous regimens to ARV of different mechanism (Group 2). The two groups differed significantly in the proportion of injecting drug users (IDU) (Group 1 vs Group 2, 10.9 vs 26.1%) and median PVL (67 vs 159 copies/mL), and the proportion of PVL<200 copies/mL (84.0% vs 58.7%) when low-level viremia was first detected. In Group 1, 39 (32.8%) continued two nucleoside reverse-transcriptase inhibitors (NRTIs) plus nNRTI; 29 (24.4%) 2 NRTIs plus PI, 47 (39.5%) 2 NRTIs plus boosted PI, and 4 (3.3%) 2 NRTIs plus integrase inhibitor (II). In Group 2, two (4.3%) switched to 2 NRTIs plus PI, 38 (82.6%) 2 NRTIs plus boosted PI, three (6.5%) 2 NRTIs plus II and three (6.5%) boosted PI plus II. In multivariate analysis, IDUs (adjusted odds ratio [AOR], 6.757; 95% CI 2.427–18.868) and PVL of 200–999 copies/mL at enrollment (AOR, 4.902; 95% CI 1.992–12.048) were more likely to be switched. At 48 weeks, patients in Group 2 were more likely to achieve PVL<40 copies/mL than Group 1 (82.6% vs 63.0%, p=0.016), while no difference was observed in achieving PVL <200 copies/mL between the two groups (95.7% vs 92.4%, p=0.729).

**Conclusions:**

According to the clinical guidelines of BHIVA, patients with low-level viremia who switched to cART consisting of 2 NRTIs plus boosted PI or newer mechanisms were more likely to re-establish viral suppression to <40 copies/mL at week 48.

**Table 1:** Characteristics of patients with persistent low level viremia

	Total (n=165)	No switch of cART (n=119)	Switch of cART (n=46)	p
Age±SD (y/o)	39±11	38±11	41±10	0.096
Male (%)	158 (95.8%)	114 (95.8%)	44 (95.7%)	0.967
MSM (%)	122 (73.9%)	91 (76.5%)	31 (67.4%)	0.242
IDU (%)	25 (15.2%)	13 (10.9%)	12 (26.1%)	0.027
HBV (%)	19 (11.5%)	14 (11.8%)	3 (6.5%)	0.810
HCV (%)	28 (17.0%)	17 (14.3%)	9 (19.6%)	0.330
Before cART CD4, median (range), cells/uL	184 (1–928)	187 (1–707)	171 (10–928)	0.937
Before cART PVL, median, copies/mL, log_10_	5.29	5.29	5.29	0.312
PVL>5 log_10_ (%)	98 (59.4%)	73 (61.3%)	25 (54.3%)	0.480
CD4 at LLV, median (range), cells/uL	422 (63–1092)	419 (63–1092)	431 (134–1010)	0.156
PVL at enrollment, copies/mL	83 (21–999)	67 (21–939)	159 (21–999)	0.002
PVL of 20–199 (%)	127 (77.0%)	100 (84.0%)	27 (58.7%)	0.001
PVL of 200–999 (%)	38 (23.0%)	19 (16.0%)	19 (41.3%)	0.001
Duration of cART exposure, median (range), weeks	47 (12–391)	44 (15–321)	58 (12–391)	0.062
Years of HIV diagnosed, median, (range), years	4 (1–14)	4 (1–14)	4 (2–13)	0.356
Ever treatment failure (%)	42/165	26/119	16/46	0.111
Current cART				
2NRTIs+nNRTI	39 (23.6%)	39 (32.8%)	0 (0%)	<0.001
2NRTIs+PI	31 (18.8%)	29 (24.4%)	2 (4.3%)	0.003
2NRTIs+PI/r	85 (51.5%)	47 (39.5%)	38 (82.6%)	<0.001
2NRTIs+II	7 (4.2%)	4 (3.3%)	3 (6.5%)	0.967
II+PI/r	3 (1.8%)	0 (0%)	3 (6.5%)	0.021
